# Gentamicin-Loaded Electrospun PVA/Kefiran/Schizophyllan Membrane for Skin Tissue Engineering Applications

**DOI:** 10.3390/polym18131679

**Published:** 2026-07-07

**Authors:** Karla Katiushka Solís-Arévalo, Luis J. Galán-Wong, Aida Rodriguez-Garcia, Katiushka Arévalo-Niño

**Affiliations:** Universidad Autónoma de Nuevo León, Facultad de Ciencias Biológicas, Instituto de Biotecnología, Ciudad Universitaria, Av. Pedro de Alba S/N, San Nicolás de los Garza C.P. 66455, Nuevo León, Mexico; karla.solisarv@uanl.edu.mx (K.K.S.-A.); luis.galanwn@uanl.edu.mx (L.J.G.-W.); aida.rodriguezgrc@uanl.edu.mx (A.R.-G.)

**Keywords:** electrospinning, skin tissue engineering, wound dressing, nanofibers, biopolymer, drug delivery

## Abstract

Healthcare-associated infections are prevalent in hospitals, clinics, and long-term care facilities. The use of wound dressings on active skin wounds, like burns, can cause damage to the skin barrier when removed for cleaning. Electrospun biodegradable and biocompatible membranes have emerged as promising alternatives for wound dressing applications. In the present study, an electrospun membrane composed of polyvinyl alcohol/kefiran/schizophyllan loaded with gentamicin and ascorbic acid was developed. Kefiran was obtained from kefir with a 0.61% extraction yield. Beadless electrospun membranes with a diameter of 400 nm were obtained. Antimicrobial activity of the membrane against *Staphylococcus aureus* and *Pseudomonas aeruginosa* was determined. Growth inhibition halos of 16.4 ± 2.2 mm were found for *Pseudomonas aeruginosa*. Furthermore, the membrane cytocompatibility assay of the membrane showed no cell toxicity in human dermal fibroblasts (HDFn cells). The produced membranes showed potential to be used as a wound dressing material in the future.

## 1. Introduction

Healthcare-associated infections (HAIs) are infections that are acquired by patients during their medical care within a healthcare facility [1]. These infections can occur in various healthcare settings, including hospitals, long-term care facilities, and outpatient clinics. HAIs can be caused by a diverse array of microorganisms, including bacteria, viruses, fungi, and parasites. Currently, HAIs remain a persistent challenge, leading to increased morbidity and mortality among patients, prolonging hospital stays, amplifying patient discomfort and suffering, and escalating healthcare costs [2,3,4]. In developed nations, HAIs affect 7% of the patient population, while in developing countries, this figure rises to about 10%. According to a report from the Centers for Disease Control and Prevention (CDC), it is estimated that in the United States, around 1.7 million cases of HAIs result in approximately 99,000 deaths annually [5]. These infections not only pose significant challenges to patients but also to healthcare providers, who must allocate additional resources and effort for effective infection control and prevention [6]. Additionally, HAIs can contribute to the growing problem of antibiotic resistance, complicating the successful treatment of infections. Several of the most significant pathogens linked to HAIs (including methicillin-resistant *Staphylococcus aureus*, vancomycin-resistant enterococci, *Acinetobacter baumannii*, *Klebsiella pneumoniae*, *Escherichia coli*, *Pseudomonas aeruginosa*, *Enterobacter* species, and *Clostridioides difficile*) [7]. For active skin wounds, like burns, *Staphylococcus aureus* and *Pseudomonas aeruginosa* are the most prevalent bacteria causing these infections [8].

Infection prevention strategies for burn wounds commonly include the use of antiseptics and wound dressings to reduce contamination and promote tissue repair [9].

However, these types of materials require change and disposal after a few hours, which in turn can lead to damage to the skin barrier when removed. One alternative to conventional passive wound dressings is the development of advanced biomaterial-based wound dressings with enhanced biological functionality.

Biomaterials refer to substances or a mixture of several substances, excluding drugs, that are employed to enhance or substitute, either partially or completely, any tissue, organ, or bodily function for an extended duration, with the aim of sustaining or enhancing an individual’s quality of life [10]. These materials can be derived from either synthetic or natural sources. Different fabrication methods have been explored for the development of biomaterial-based scaffolds for skin tissue engineering applications, including solvent casting, freeze-drying, phase separation, hydrogel formation, and 3D bioprinting [11,12,13,14]. Among these techniques, electrospinning has attracted particular attention because it enables the production of nano- and microfibrous structures [15] that mimic the architecture of the extracellular matrix [13]. Over the last few years, electrospinning has garnered interest for its use in wound healing applications, primarily because of its simple methodology, cost-effectiveness and reproducibility [16].

Polyvinyl alcohol (PVA) is a synthetic polymer that has been extensively studied for biomedical and tissue engineering applications as a base polymer due to its favorable biocompatibility, hydrophilic character, water solubility, and ability to be electrospun efficiently [17,18,19]. Furthermore, PVA has been widely applied in wound healing and tissue engineering systems due to its capacity to produce homogeneous nanofibers and its compatibility with natural biopolymers [20,21,22].

Natural biopolymers have emerged as essential materials in tissue engineering due to their inherent biocompatibility, biodegradability, and bioactivity. Among them, kefiran and schizophyllan have attracted considerable attention for biomedical applications [20,23,24,25,26,27,28,29,30,31].

Kefiran is an exopolysaccharide (EPS) composed primarily of glucose and galactose [21,22], synthesized by microorganisms present in kefir grains—a symbiotic microbial community of lactic acid bacteria, yeasts, and acetic acid bacteria [32,33,34,35,36,37,38,39]. It exhibits a range of beneficial biological properties, including antimicrobial, anti-inflammatory, and immunomodulatory effects, which make it suitable for applications in wound healing and tissue regeneration [26]. Additionally, kefiran demonstrates good film-forming ability and a gel-like consistency, allowing it to be integrated into various delivery systems and scaffolds.

Similarly, schizophyllan is another structurally unique EPS produced by the white-rot fungus *Schizophyllum commune* [40]. It consists of a β-(1→3)-D-glucan backbone with β-(1→6)-linked side chains, a configuration that contributes to its stability and bioactivity [41]. Schizophyllan has been shown to promote wound healing by enhancing macrophage activation, promoting fibroblast proliferation, and supporting extracellular matrix formation [42,43]. Its excellent water retention capacity and ability to form viscous solutions also make it a strong candidate for use in hydrogel and membrane-based systems for controlled drug delivery and tissue engineering applications [44,45]. Furthermore, chemical agents such as ascorbic acid act as an antioxidant and can stimulate collagen synthesis, crucial for tissue repair and reduction in oxidative stress injuries [46].

While kefiran and schizophyllan have individually demonstrated promising properties for wound healing applications, their combination within a single electrospun scaffold has not been previously reported. Kefiran contributes biocompatibility properties [26,47], whereas schizophyllan provides water retention capacity and bioactivity associated with fibroblast proliferation [20,48]. Therefore, combining both polysaccharides may offer complementary biological functions while maintaining favorable electrospinning characteristics.

The present study aimed to develop and characterize a biocompatible electrospun membrane based on kefiran and schizophyllan biopolymers incorporating bioactive agents to create a multifunctional biodegradable scaffold with antimicrobial activity against *S. aureus* and *P. aeruginosa* and potential applicability in wound healing.

## 2. Materials and Methods

### 2.1. Materials

Whole cow’s ultrapasteurized milk (Lala^®^, Torreón, México) was purchased from a local supermarket. Ethanol 96% (Jalmek Científica S.A. de C.V., San Nicolás de los Garza, México) was used for polymer extraction. Mueller-Hinton broth and agar (Difco^®^) were purchased from CTR Scientific, México (Mexico City, Mexico). Polyvinyl alcohol (Airvol 540) was purchased from Air Products and Chemicals, Inc (Allentown, PA, USA). Gentamicin (Pharmalife^®^) was purchased from a local drug store in Monterrey, Mexico. Ascorbic acid (Fermont^®^) was purchased from Productos Químicos Monterrey S.A. de C.V., México (Monterrey, Mexico). *Staphylococcus aureus* BAA-44 and *Pseudomonas aeruginosa* ATCC 27853 were purchased from the American Type Culture Collection (Manassas, VA, USA).

### 2.2. Kefiran Extraction

Kefir grains were maintained in cow’s milk for the duration of the experiment. Every two days, the grains were carefully rinsed with distilled water and added to fresh media. For the EPS extraction, two extraction methodologies were evaluated to determine the most suitable procedure for kefiran recovery and subsequent membrane fabrication. The first procedure was carried out as Piermaria et al. [49] with some modifications. Briefly, kefir grains were added to distilled water at a 10:1 ratio of water–kefir and then heated to 100 °C for 1 h. Then, the solution was centrifuged at 6500 rpm for 20 min at 25 °C and the supernatant was mixed with cold ethanol and left at −20 °C overnight. The next day, the solution was centrifuged at 6500 rpm for 20 min at 4 °C and the pellet was washed. This step was performed twice. The obtained EPS was then freeze-dried and stored in a desiccator chamber until used.

For the second methodology, the procedure was carried out as Hasheminya et al. [50] with some modifications. In short, kefir grains were added to distilled water at a 10:1 ratio of water–kefir and then heated in a metabolic water bath at 50 °C for 10 min. Then, the solution was treated with ultrasound at 50 kHz for 10 min. Finally, the solution was centrifuged and alcohol precipitated as described previously.

### 2.3. Schizophyllan Extraction

The production and extraction of schizophyllan were performed following previously reported methodologies with minor modifications [51,52,53]. In brief, five 6 mm-diameter mycelial discs from a Petri dish containing *Schizophyllum commune* ScIBL1, provided by Laboratory L1 of the Institute of Biotechnology, FCB-UANL, were added to 100 mL of yeast extract, peptone, and dextrose (YPD) media. The flasks were incubated at 30 °C and agitated at 150 rpm for 7 days. After the incubation period, cultures were centrifuged at 8000 rpm for 20 min at 5 °C to separate the supernatant from the fungal biomass. The supernatant was collected, and schizophyllan was precipitated using absolute ethanol in a 1:2 (*v*/*v*) ratio. The resulting precipitate was filtered and dried in a furnace at 70 °C for 24 h, then ground into powder using a ceramic mortar. The polymer powder was then stored in a dry, sealed container until further use.

### 2.4. Gentamicin Minimum Inhibitory Concentration

The minimum inhibitory concentration (MIC) of gentamicin against *S. aureus* and *P. aeruginosa* was determined with the broth microdilution method [54], where 20 μL of a 10^8^ CFU mL^−1^ culture was added to a sterile 96-well plate. Gentamicin was added in concentrations of 1.25, 2.5, 5, 10, 20, 40 and 80 mgmL^−1^. Optical density (OD) was measured at 595 nm using an EZ Read 2000 microplate reader (Biochrom, Cambridge, UK) and read again after 24 h of incubation at 37 °C. The MIC was determined as the lowest antibiotic concentration at which the OD at 24 h of inoculum remained the same or decreased compared to the initial reading [55,56].

### 2.5. Electrospinning of Membranes

Two preparations were made as a control for the electrospinning process. The first polymer solution was prepared with 8% polyvinyl alcohol (PVA) in distilled water; this solution was used as the guide polymer control and was called SP. The second polymer solution consisted of a preparation of 8% PVA, 1% schizophyllan and 1% kefiran in distilled water; this was called SPKS.

Finally, a solution of 8% PVA/1% schizophyllan/1% kefiran in distilled water was prepared, and a concentration of 30.42 μM of ascorbic acid was added. Finally, an addition of six times more of the MIC of gentamicin determined previously was made. This is due to a previous study where a loss of activity was observed after the electrospinning process [53]. This last solution was called SPKS-GA. The polymeric solutions and membranes obtained can be summarized in Table 1.

**Figure 1:**
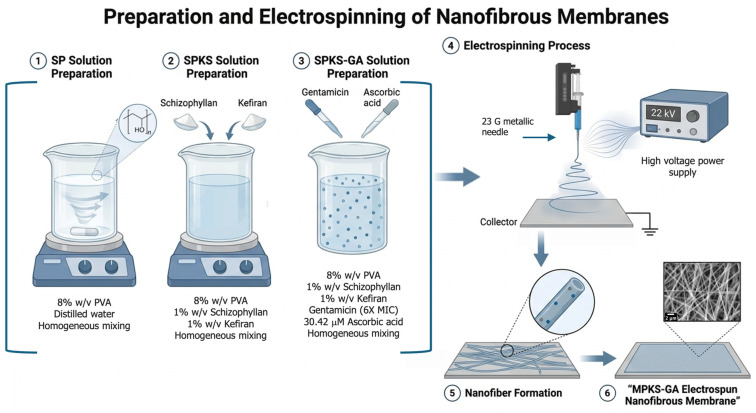
Schematic representation of the preparation of SP, SPKS, and SPKS-GA solutions and the subsequent electrospinning process used to fabricate nanofibrous membranes. Numbers 1–6 indicate the sequential steps of the process.

The main conditions that influence the electrospinning process were tested in a Standard Unit NEU-01 (Tong Li Tech Co., Shenzhen, China) using a metallic plate collector and a 23 G (0.6 mm diameter) needle. For this, a 2^3^ factorial design with a central point was applied to determine the effective electrospinning parameters [53]. In this factorial design, the following were varied: flow rate (0.5–2.0 mLh^−1^), distance to collector (10–20 cm), and applied voltage (5–20 kV) according to what has been reported in the literature to obtain membranes with fibers with a diameter in the nano or micrometric scale with the solutions developed previously. This process is represented in Figure 1. Subsequently, membranes in which the characteristics of fibers with smooth surfaces without the presence of drops or “beads”, which are the desired morphology, were further studied.

### 2.6. Conductivity of Solutions

The electrical conductivity of the electrospinning solutions was measured at room temperature using a Pinnacle 542 conductivity meter (Corning, Somerville, MA, USA) equipped with a conductivity probe. Prior to analysis, the solutions were gently homogenized to ensure uniformity. Conductivity measurements were performed in triplicate for each formulation (MP, MPKS, and MPKS-GA), and the average values were recorded in µS/cm.

### 2.7. Fiber Morphology

A primary evaluation of the morphology of the fabricated membranes was performed using optical microscopy. Selected membranes were then morphologically determined using scanning electron microscopy on an EVO MA25 microscope (Zeiss, Jena, Germany) at 15 kV. Fiber diameter analysis was performed using ImageJ software version 1.53a by measuring a minimum of 100 randomly selected fibers from ten representative images.

### 2.8. Water Contact Angle

In order to determine the hydrophilicity level of the membranes, a water contact angle study was performed. The evaluation was carried out using an OCA 15 Plus Contact Angle device (Dataphysics Instruments, Filderstadt, Germany) and SCA 20 software to determine the measurements. In brief, the sample was placed in the sample holder plate at a temperature of 25 °C. A drop of deionized water of pre-established volume was placed on the sample, to immediately take a video of it. Subsequently, using the SCA 20 software, the contact angle between the sample and the internal part of the drop was calculated.

### 2.9. Infrared Spectroscopy Analysis of Membranes

Membrane analysis was performed by Fourier transform infrared spectroscopy (FTIR) in a range of 4000 to 400 cm^−1^ using the Nicolet iS10 (Thermo Fischer Scientific, Waltham, MA, USA) with an ATR attachment at a resolution of 4 cm^−1^ at 64 scans with baseline adjustment.

### 2.10. Antimicrobial Activity of Membranes

The evaluation of the antimicrobial activity of the membranes against *S. aureus* and *P. aeruginosa* was carried out using the agar diffusion technique as described by the American Society of Microbiology [57]. First, the microorganisms were adjusted to the 0.5 Mc Farland scale. Subsequently, 0.1 mL of each bacterium was inoculated into Petri dishes with Mueller-Hinton agar. Then, 6 mm circles of the membranes were placed in the agar. Sterile Whatman no. 40 filter paper loaded with 10 μL of antibiotic (gentamicin) was used as a positive control, and distilled water as a negative control. The plates were incubated at 37 °C for 24 h, and the inhibition zone was measured [58].

### 2.11. Cell Viability

Cell viability was analyzed using the MTT assay. In short, human dermal fibroblasts (HDFn cells) were cultured in Dulbecco’s Modified Eagle (DMEM) medium supplemented with 10% fetal bovine serum and 1% antibiotic/antimycotic, maintained at 37 °C, ≈85% relative humidity, and in an atmosphere of 95% air with 5% CO_2_. For the test, 5000 cells/well were seeded in 96-well plates, and after 24 h of adhesion, they were exposed to varying concentrations (25%, 50%, 75%, and 100%) of sterilized membranes for 24, 48, and 72 h to assess cytotoxicity. Subsequently, 20 µL of MTT was added, incubating for 2 h, followed by removal of the medium and the addition of 100 µL of Dimethyl sulfoxide to solubilize the crystals, reading the absorbance at 570 nm in an EZ Read 2000 microplate reader (Biochrom, Cambridge, UK). All tests were performed in triplicate to ensure statistical reliability. Results were analyzed to determine the impact of membrane concentration and exposure time on cell viability.

### 2.12. Statistical Analysis

Statistical analyses were made by conducting a one-way ANOVA using SPSS Statistics 25 (IBM, Armonk, NY, USA). Differences were considered significant if *p* < 0.05. A Tukey test was carried out as a post hoc analysis.

## 3. Results

### 3.1. Kefiran Extraction

Kefiran was extracted using two different heating methodologies: a conventional hot extraction at 100 °C and a mild heating extraction at 50 °C coupled with ultrasound treatment, both followed by ethanol precipitation.

The extraction yield obtained with the hot method (100 °C) was 0.39%. In contrast, the mild heat plus ultrasound method resulted in a higher yield of 0.61%. Therefore, the ultrasound-assisted extraction increased kefiran recovery under the evaluated conditions.

Representative photographs of the kefir grains before and after the ultrasound-assisted extraction process are presented in Supplementary Figure S1.

Macroscopic observation revealed noticeable structural changes after treatment. Untreated grains displayed a well-defined granular morphology, whereas treated grains exhibited a softer, more hydrated, and more homogeneous appearance, with partial loss of their characteristic spherical structure. These observations are qualitative and are based on visual inspection of the grains. However, after two days of incubation in fresh culture medium, the grains recovered their original shape.

### 3.2. Schizophyllan Extraction

On day 7 of incubation, the defined YPD medium supported schizophyllan production reaching a concentration of 1.97 ± 0.33 g L^−1^. This time point also corresponded to the highest biomass concentration, which reached 1.53 g L^−1^.

The simultaneous occurrence of maximum polysaccharide production and peak biomass suggests a relationship between fungal growth and schizophyllan synthesis under these culture conditions.

### 3.3. Gentamicin Minimum Inhibitory Concentration

The MIC of gentamicin was determined by broth microdilution against *Staphylococcus aureus* BAA-44 (Methicillin-resistant *Staphylococcus aureus*) and *Pseudomonas aeruginosa* ATCC 27853. The MIC values obtained were 100 μg/mL for *S. aureus* and 200 μg/mL for *P. aeruginosa*.

To compensate for potential antimicrobial activity loss during the electrospinning process, the highest MIC value (200 μg/mL) was increased sixfold prior to incorporation into the polymeric formulations.

### 3.4. Membrane Electrospinning

According to the experimental design, the electrospinning conditions summarized in Table 2 were evaluated to determine their effect on fiber formation using the SPKS solution.

At a working distance of 10 cm, fiber formation was not observed at 5 or 12.5 kV regardless of the applied flow rate (0.5, 1.2, or 2.0 mL h^−1^). Fiber formation was achieved at 20 kV for all three flow rates; however, in all cases, the collected structures presented abundant bead formation.

To improve fiber morphology, subsequent experiments were conducted at an increased needle-to-collector distance of 20 cm and a fixed voltage of 22 kV, while varying the flow rate from 0.1 to 0.3 mL h^−1^. Under these conditions, continuous fibers were obtained in all cases. Nevertheless, bead formation was observed at flow rates of 0.2 and 0.3 mL h^−1^. Bead-free fibers were only obtained at a flow rate of 0.1 mL h^−1^.

### 3.5. Conductivity of Solutions

The conductivity of the electrospinning solutions increased progressively with the incorporation of kefiran and bioactive compounds. The MP formulation exhibited a conductivity of 462 µS/cm, whereas the addition of kefiran in the MPKS solution increased conductivity to 850 µS/cm. The highest conductivity value was observed for MPKS-GA, reaching 1150 µS/cm. Compared with MP, the conductivity of MPKS increased by approximately 84%, while MPKS-GA exhibited an increase of nearly 149%. These results indicate that both kefiran and the incorporated bioactive compounds significantly modified the electrical properties of the precursor solutions.

### 3.6. Fiber Morphology

**Figure 2:**
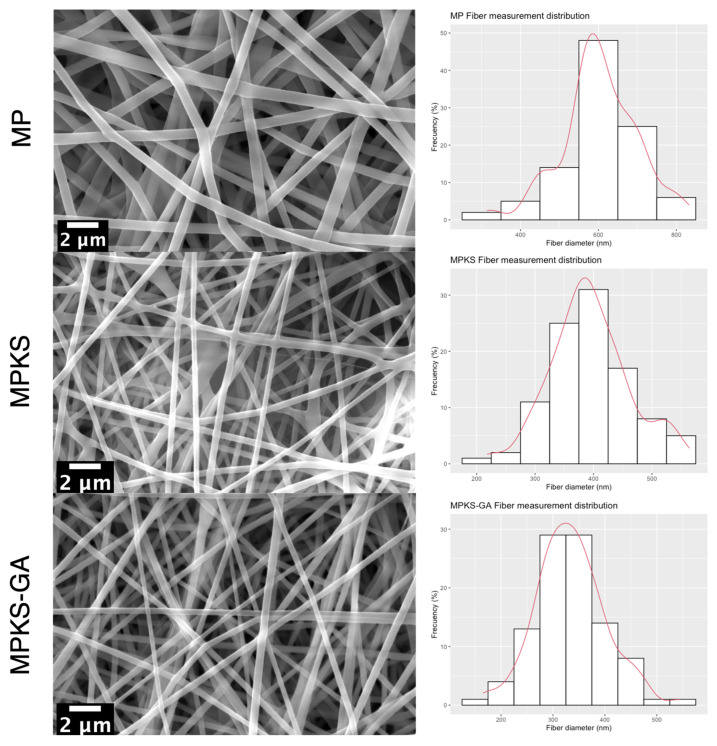
Scanning electron microscopy (SEM) images of electrospun MP, MPKS, and MPKS-GA membranes at 5000× magnification and their corresponding fiber diameter distribution histograms. The red line represents the density distribution curve of fiber diameters.

The morphology of MP, MPKS and MPKS-GA membranes is shown in Figure 2. All formulations mentioned exhibited uniform fibrous structures without the presence of beads or solution clusters, indicating stable electrospinning conditions.

Fiber diameter analysis revealed that MP fibers had an average diameter of 603 ± 97 nm, whereas MPKS fibers presented a reduced diameter of 394 ± 68 nm. The smallest fiber diameter was observed for MPKS-GA membranes, with an average thickness of 334 ± 67 nm.

Fiber diameter distributions obtained from the analysis of 100 individual fibers per formulation are presented in the corresponding histograms (Figure 2). MP fibers exhibited a broader diameter distribution, ranging from approximately 314 to 833 nm, with most fibers concentrated between 500 and 700 nm. In contrast, MPKS fibers showed a narrower distribution, predominantly between 300 and 500 nm, indicating a more homogeneous fiber population. The incorporation of gentamicin and ascorbic acid in the MPKS-GA formulation further shifted the distribution toward smaller diameters, with most fibers ranging from 200 to 400 nm. Although some larger fibers were observed, the overall distribution remained centered at lower diameter values compared to MP and MPKS. These results demonstrate a progressive reduction in fiber diameter and a shift toward finer fiber populations following the incorporation of kefiran and bioactive compounds.

### 3.7. Water Contact Angle

Contact angle measurements were performed to evaluate the hydrophilicity or hydrophobicity of the electrospun membranes. The MP membrane exhibited contact angles of 89.9 ± 28.61° (left) and 89.76 ± 28.47° (right). Similarly, MPKS membranes showed angles of 88.12 ± 8.76° on both sides.

**Figure 3:**
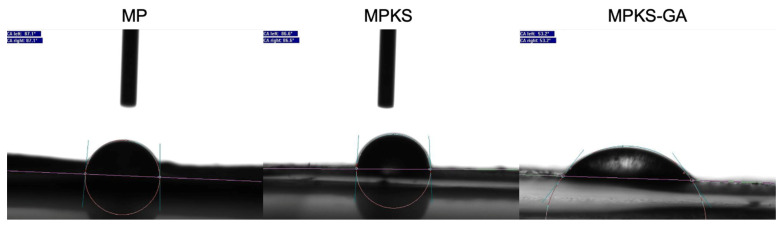
Representative water contact angle images of electrospun MP, MPKS, and MPKS-GA membranes. The colored lines indicate the droplet profile and baseline used for contact angle determination.

In contrast, MPKS-GA membranes presented significantly lower contact angles, with values of 53.61 ± 6.64° (left) and 53.64 ± 6.83° (right). Representative images are shown in Figure 3.

### 3.8. Infrared Spectroscopy Analysis of Membranes

**Figure 4:**
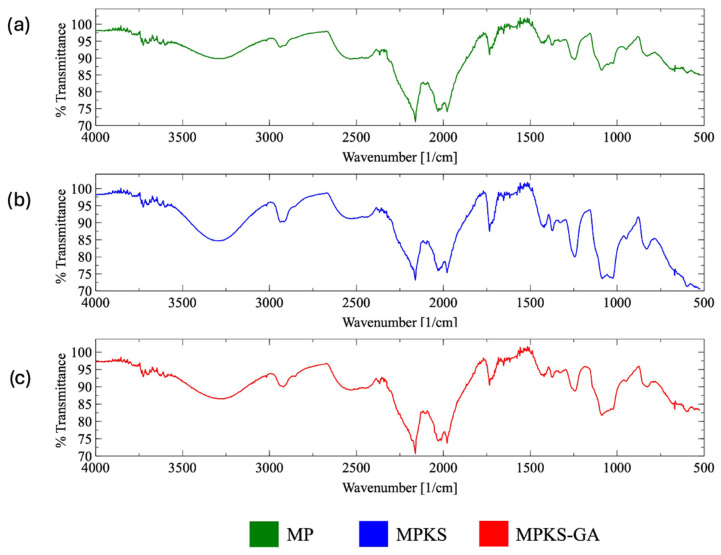
FTIR spectra of (**a**) MP (green), (**b**) MPKS (blue), and (**c**) MPKS-GA (red).

The FTIR spectra of the MP, MPKS, and MPKS-GA membranes (Figure 4) showed differences that can be attributed to the incorporation of polysaccharides and bioactive compounds.

In the MP sample, a broad band was identified in the 3200–3400 cm^−1^ region corresponding to O–H stretching vibrations associated with the hydroxyl groups of PVA and hydrogen-bonding interactions. Signals around 2900 cm^−1^ were attributed to C–H stretching vibrations of the polymer chains, while bands between 1000 and 1200 cm^−1^ corresponded to C–O stretching vibrations characteristic of the alcohol groups present in PVA.

In the MPKS sample, the broad O–H band was maintained, with slight variations in intensity and shape compared to MP. Additionally, an increase in band intensity was observed in the 1000–1100 cm^−1^ region, which can be attributed to C–O–C stretching vibrations characteristic of glycosidic linkages. These spectral changes are consistent with the presence of kefiran and schizophyllan within the PVA matrix.

The MPKS-GA membrane exhibited additional spectral changes compared to MPKS. Variations were observed in the 1600–1750 cm^−1^ region, which may be associated with carbonyl (C=O) and/or amide-related vibrations, as well as changes in the intensity of the O–H band and the 1000–1200 cm^−1^ region. These differences are consistent with the incorporation of gentamicin and ascorbic acid into the polymer matrix.

### 3.9. Antimicrobial Activity of Membranes

The antimicrobial activity of the developed membranes against *Staphylococcus aureus* BAA-44 and *Pseudomonas aeruginosa* ATCC 27853 was evaluated using the Kirby–Bauer disk diffusion method.

Among the tested formulations, SPKS-GA exhibited the highest antimicrobial activity, producing inhibition halos of 27.2 ± 0.6 mm against *P. aeruginosa* and 5.3 ± 4 mm against *S. aureus*. MPKS-GA also demonstrated antimicrobial activity against *P. aeruginosa*, with inhibition halos of 16.4 ± 2.2 mm.

In contrast, untreated membranes and their corresponding precursor solutions (MPKS, MP, SPKS, and SP) did not show inhibition halos against either microorganism.

Free gentamicin was included as a positive control in the antimicrobial assay. Free gentamicin produced inhibition halos of 34.3 ± 1.8 mm against *P. aeruginosa* and 17.0 ± 1.1 mm against *S. aureus*. As expected, the free antibiotic produced the largest inhibition zones against both microorganisms. Although the inhibition halos generated by MPKS-GA were smaller than those produced by free gentamicin, antibacterial activity against *P. aeruginosa* was retained after incorporation of the antibiotic into the electrospun membrane.

A one-way ANOVA was conducted to evaluate the influence of microorganism type (*S. aureus* vs. *P. aeruginosa*) and treatment type (MPKS-GA, MPKS, MP, SPKS-GA, SPKS, and SP) on antimicrobial activity. Significant differences were observed for microorganism type (F_1,107_ = 280.722, *p* < 0.01), treatment type (F_6,107_ = 122.974, *p* < 0.01), and their interaction (F_6,107_ = 33.871, *p* < 0.01).

### 3.10. Cell Viability

**Figure 5:**
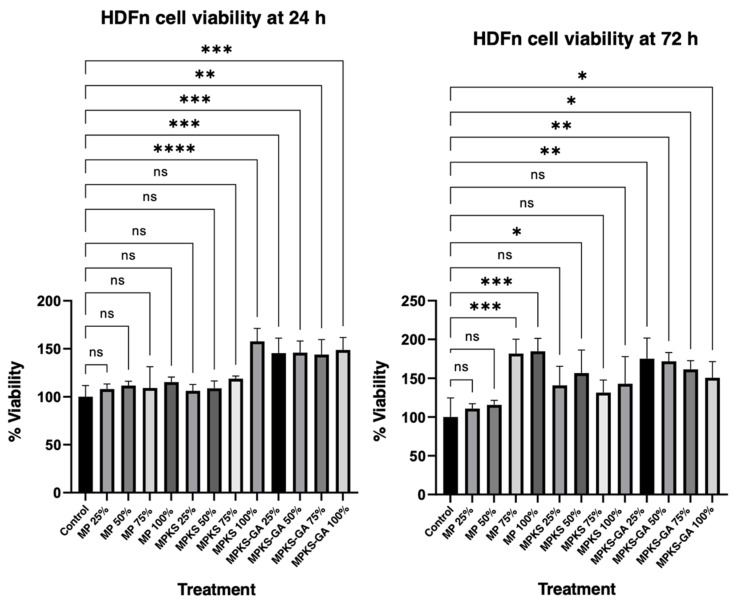
Viability of HDFn human dermal fibroblasts following exposure to different concentrations of MP, MPKS, and MPKS-GA membranes for 24 and 72 h, as determined by the MTT assay. Asterisks indicate statistically significant differences determined by ANOVA followed by Tukey’s post hoc test (* *p* < 0.05, ** *p* < 0.01, *** *p* < 0.001, and **** *p* < 0.0001); ns indicates a non-significant difference.

The effect of the electrospun membranes on the viability of HDFn cells was evaluated at 24 and 72 h (Figure 5).

At 24 h, exposure to the full MPKS membrane resulted in a significant increase in HDFn cell viability. All treatments with the MPKS-GA membrane also induced a significant increase in cell viability, indicating the absence of cytotoxic effects.

At 72 h, MP membranes significantly increased cell viability when applied at 75% and 100% membrane coverage. Similarly, MPKS membranes enhanced cell viability when used at 50% coverage. Notably, MPKS-GA treatment significantly increased HDFn cell viability at all tested concentrations.

Overall, none of the membranes exhibited cytotoxic effects at either time point.

## 4. Discussion

### 4.1. Kefiran Extraction

The higher extraction yield obtained with the mild heat and ultrasound method suggests that ultrasonic treatment may enhance polysaccharide release from the kefir grain matrix. Ultrasound has been reported to improve mass transfer and facilitate the disruption of intermolecular interactions, potentially promoting the release of extracellular polysaccharides. Previous studies have used ultrasound frequencies ranging from 24 to 37 kHz for polysaccharide extraction [50,59,60]. In this study, a frequency of 50 kHz was applied, which may have influenced the extraction efficiency. Although different ultrasonication conditions have been associated with variations in polysaccharide yield [60], the exact mechanism underlying this effect remains unclear.

It is important to note that the biopolymer extraction yields can vary depending on several factors, including kefir grain surface area, solvent type, extraction time and temperature, as well as washing and centrifugation steps during purification [61,62,63]. Therefore, differences between the present results and those reported in the literature may be attributed to methodological variations.

Although the extraction yield obtained in this study was lower than some previously reported values [26,50,64,65], an important advantage of the proposed methodology is that the kefir grains were not destroyed during the process. Despite temporary morphological alterations, the grains recovered their original structure after re-cultivation in fresh medium. This characteristic reduces biomass consumption and allows repeated extractions, which is particularly advantageous when working with limited amounts of kefir grains.

### 4.2. Schizophyllan Extraction

The results indicate that schizophyllan production in YPD medium is closely associated with fungal growth, as both maximum biomass and the highest polymer concentration were observed on day 7. This suggests that schizophyllan biosynthesis may be partially growth-associated under the evaluated conditions.

The defined composition of the YPD medium likely contributed to consistent nutrient availability, promoting sustained metabolic activity in *Schizophyllum commune* and favoring polysaccharide accumulation in the culture supernatant [66]. Readily assimilable carbon and nitrogen sources present in YPD may enhance primary metabolism, which in turn can support extracellular polysaccharide production.

These findings are consistent with reports indicating that nutrient-rich defined media can positively influence schizophyllan production by maintaining stable growth kinetics and metabolic flux toward polysaccharide biosynthesis [67,68,69,70,71].

### 4.3. Gentamicin Minimum Inhibitory Concentration

The MIC obtained for *S. aureus* BAA-44 (100 μg/mL) reflects the reduced susceptibility typically associated with Methicillin-resistant *Staphylococcus aureus* (MRSA) strains. Although this strain is reported as gentamicin-resistant [72], measurable growth inhibition was observed at the tested concentration, suggesting partial susceptibility under the experimental conditions employed. Variability in reported MIC values for MRSA strains is well documented. For instance, previous studies have shown that gentamicin alone failed to inhibit *S. aureus* BAA-44 at 150 μM unless combined with a 2-aminoimidazole compound, indicating that synergistic strategies may be required to overcome resistance mechanisms [73].

Comparatively, substantially lower MIC values have been reported for non-MRSA strains, such as *S. aureus* ATCC 12600, particularly when gentamicin is delivered via controlled-release systems [74]. Conversely, other MRSA strains (*S. aureus* ATCC 43300) have demonstrated MIC values as high as 1 mg/mL, underscoring the strong strain-dependent variability in aminoglycoside susceptibility [75].

For *P. aeruginosa* ATCC 27853, the MIC obtained in this study (200 μg/mL) falls within the broad range reported in the literature (0.031 μg/mL to 0.500 mg/mL) [75,76]. This wide dispersion likely reflects differences in assay methodology, inoculum density, growth phase, and laboratory-specific testing conditions. Moreover, intrinsic resistance mechanisms in *P. aeruginosa*, including efflux pumps and reduced outer membrane permeability, may contribute to elevated MIC values.

### 4.4. Membrane Electrospinning

The results demonstrate that voltage, working distance, and flow rate significantly influence fiber formation and morphology during electrospinning of the SPKS solution.

At a 10 cm working distance, increasing the voltage to 20 kV enabled jet formation and fiber collection; however, the presence of abundant beads suggests insufficient jet stretching and/or incomplete solvent evaporation before reaching the collector. High flow rates likely contributed to bead formation by supplying excess solution that could not be fully elongated under the applied electric field.

Increasing the needle-to-collector distance to 20 cm and voltage to 22 kV improved fiber formation, likely attributed to enhanced jet stretching and longer flight time, which favors solvent evaporation and fiber solidification. Under these conditions, reducing the flow rate minimized bead formation, with 0.1 mL h^−1^ producing uniform, bead-free fibers. This indicates that lower flow rates promote greater jet stability and allow sufficient time for solvent evaporation, resulting in improved fiber morphology [77].

Overall, the optimized electrospinning parameters for bead-free fiber formation were 22 kV, 20 cm working distance, and 0.1 mL h^−1^ flow rate.

### 4.5. Conductivity of Solutions

The increase in conductivity observed in the MPKS formulation compared to MP may be attributed to the presence of ionizable functional groups and residual ionic species associated with kefiran [31,78] as well as to the overall changes in solution composition resulting from the incorporation of the polysaccharides. Furthermore, the incorporation of gentamicin and ascorbic acid in MPKS-GA contributed to a further increase in conductivity due to their ability to form ionic species in aqueous solutions and their ability to increase charge carrier concentration within the solution [79,80]. Changes in solution conductivity are known to influence electrospinning behavior by increasing charge density within the polymer jet, resulting in stronger electrostatic stretching forces during fiber formation [81,82,83]. This behavior is consistent with the morphological analysis, where a progressive decrease in average fiber diameter was observed from MP (603 ± 97 nm) to MPKS (394 ± 68 nm) and MPKS-GA (334 ± 67 nm). Therefore, the conductivity trend (MP < MPKS < MPKS-GA) likely contributed to the enhanced jet elongation and the formation of thinner fibers in the modified formulations.

### 4.6. Fiber Morphology

The reduction in fiber diameter observed for MPKS and MPKS-GA compared to the MP control suggests that incorporation of additional components modifies the physicochemical properties of the electrospinning solution. In particular, the presence of kefiran and schizophyllan in MPKS, and further incorporation of gentamicin and ascorbic acid in MPKS-GA, likely altered solution viscosity, conductivity, and intermolecular interactions, leading to enhanced jet stretching.

The smallest fiber diameter obtained in MPKS-GA membranes may be attributed to the addition of ascorbic acid. Previous reports have shown that incorporation of ascorbic acid into polymeric electrospinning solutions can reduce fiber diameter. For example, electrospun membranes composed of poly(L-lactic acid)-co-poly-(ε-caprolactone), silk fibroin, tetracycline hydrochloride, and ascorbic acid showed a decrease in fiber diameter from 315 ± 45 nm (without ascorbic acid) to 265 ± 97 nm (with ascorbic acid) [80].

Furthermore, the fiber diameters obtained in this study fall within or below the range reported for similar systems. PVA/kefiran fibers have been reported with diameters ranging from 305 ± 35 to 766 ± 43 nm [22], while PVA/schizophyllan fibers have shown diameters between 353 ± 84 and 589 ± 84 nm [20]. The comparatively smaller diameters observed here may be attributed to the optimization of electrospinning parameters through experimental design, allowing improved control over fiber formation.

The diameter distribution analysis provides additional evidence of the influence of solution composition on fiber formation. MP fibers displayed the widest diameter distribution, suggesting greater variability during jet stretching and solidification. The addition of kefiran not only reduced the average fiber diameter but also narrowed the distribution range, indicating improved process stability and more uniform fiber formation. This effect may be associated with the increased electrical conductivity of the polymer solution, which enhances charge repulsion within the electrospinning jet and promotes more efficient elongation. A similar trend was observed for MPKS-GA, where the highest conductivity values corresponded to the smallest average fiber diameter and a distribution shifted toward finer fibers. The progressive displacement of the diameter distributions from MP to MPKS and MPKS-GA supports the hypothesis that the incorporation of kefiran, gentamicin, and ascorbic acid modified the electrospinning solution properties, leading to enhanced jet stretching and the production of thinner nanofibers [84,85]. Furthermore, the absence of beads and the diameter distributions observed for MPKS and MPKS-GA indicate that the selected electrospinning parameters were suitable for producing homogeneous fibrous membranes.

### 4.7. Water Contact Angle

The contact angle values obtained for MP and MPKS membranes (≈88–90°) indicate moderately hydrophilic behavior, which is consistent with the presence of hydrophilic polymers such as PVA, kefiran, and schizophyllan [86].

The marked reduction in contact angles for MPKS-GA membranes suggests enhanced hydrophilicity due to the incorporation of gentamicin and ascorbic acid. The presence of ascorbic acid, a polar and water-soluble compound, likely contributed to increased surface energy and improved water affinity.

Similar findings have been reported in the literature. For instance, polyacrylonitrile/kefiran electrospun fibers have shown strongly hydrophilic behavior with contact angles as low as 12.3 ± 1.13° [87]. Additionally, PVA-based electrospun membranes commonly exhibit contact angles below 50° [88,89], reflecting their hydrophilic nature. Moreover, incorporation of ascorbic acid into electrospun systems based on polylactic acid and silk fibroin has been associated with decreased contact angles and increased hydrophilicity [80].

Although surface roughness in electrospun membranes can influence hydrophilic or hydrophobic behaviors, the observed increase in hydrophilicity in MPKS-GA is likely dominated by the polar nature of ascorbic acid [90]. From a biomedical perspective, moderate surface hydrophilicity has been reported to favor cellular interactions [91,92], making such materials attractive for tissue engineering and wound healing applications.

### 4.8. Antimicrobial Activity of Membranes

The results demonstrate that antimicrobial activity was exclusively associated with the gentamicin-loaded solution and membrane (SPKS-GA and MPKS-GA), confirming the role of the incorporated antibiotic in bacterial growth inhibition. The absence of inhibition halos in untreated membranes indicates that the polymeric matrix alone does not exert intrinsic antibacterial effects under the tested conditions.

The larger inhibition halos observed against *P. aeruginosa* compared to *S. aureus* suggest differential susceptibility between Gram-negative and Gram-positive bacteria under these experimental conditions. The relatively small inhibition zone against *S. aureus* BAA-44 may be attributed to its methicillin-resistant phenotype (MRSA), which is known to exhibit reduced susceptibility to aminoglycosides.

Comparable studies have reported similar trends. For instance, Cesur et al. [80,93] described a PVA/Gelatin/Gentamicin scaffold producing inhibition halos of 16 mm against *S. aureus* and 26 mm against *P. aeruginosa*, values consistent with the antibacterial performance observed in this study.

To the best of our knowledge, no previous reports describe scaffolds composed of PVA/Kefiran/Schizophyllan for antimicrobial drug delivery. Therefore, the present findings support the novelty of this polymeric system and its potential application as an antibacterial drug-delivery membrane.

### 4.9. Cell Viability

The observed increase in HDFn cell viability suggests that the electrospun membranes are biocompatible and may actively promote fibroblast proliferation. The presence of kefiran in MPKS and MPKS-GA membranes likely contributed to this effect, as previous studies have reported that kefiran enhances cell adhesion and proliferation, which are essential processes in tissue regeneration [31].

Kefiran has also been associated with protective effects against oxidative stress, which may further support fibroblast growth and function. Additionally, previous investigations have shown that kefiran-coated electrospun polylactic acid structures improve fibroblast adhesion, proliferation, and collagen production [94].

Schizophyllan may also contribute to the observed biological response. Its triple-helix conformation has been associated with bioactivity and enhanced cell interactions [95]. Studies have reported that schizophyllan incorporated into PVA nanofibers promotes fibroblast proliferation and migration, particularly at specific polymer ratios, supporting its suitability for wound healing applications [20,86].

Therefore, the combined presence of kefiran and schizophyllan in the developed membranes may create a favorable microenvironment that supports fibroblast growth, making these materials promising candidates for regenerative and wound healing applications.

## 5. Conclusions

Electrospun polyvinyl alcohol/kefiran/schizophyllan membranes incorporating gentamicin and ascorbic acid were successfully fabricated, producing homogeneous fibers with enhanced hydrophilicity and favorable cytocompatibility. The incorporation of bioactive components reduced the water contact angle and generated membranes that supported HDFn fibroblast viability, indicating their suitability for biomedical applications.

The MPKS-GA membranes exhibited antibacterial activity against *Pseudomonas aeruginosa*, whereas no inhibitory effect was observed against *Staphylococcus aureus* under the evaluated conditions. In contrast, the precursor SPKS-GA solution inhibited the growth of both microorganisms, suggesting that incorporation into the electrospun matrix may influence antibiotic availability and diffusion.

To the best of our knowledge, this is the first report describing electrospun polyvinyl alcohol/kefiran/schizophyllan membranes simultaneously incorporating gentamicin and ascorbic acid. The developed system combines the complementary properties of kefiran and schizophyllan with the antibacterial activity of gentamicin and the bioactive potential of ascorbic acid, resulting in a multifunctional scaffold with potential application as an antimicrobial wound dressing. Further studies evaluating the crystallinity, thermal stability, mechanical properties, and release behavior of the incorporated bioactive agents are warranted to fully assess their clinical potential.

## Figures and Tables

**Table 1:** The composition of polymer solutions and the inhibition zone measured.

Solution Code	Polymer Composition	Additives	Electrospun Membrane Code
SP	8% *w*/*v* PVA in distilled water	-	MP
SPKS	8% *w*/*v* PVA, 1% *w*/*v* schizophyllan, 1% *w*/*v* kefiran in distilled water	-	MPKS
SPKS-GA	8% *w*/*v* PVA, 1% *w*/*v* schizophyllan, 1% *w*/*v* kefiran in distilled water	Ascorbic acid and gentamicin (6× MIC)	MPKS-GA

**Table 2:** Electrospinning test parameters with SPKS solution and fiber formation outcomes.

Distance (cm)	Flow (mLh^−1^)	Voltage (kV)	Fiber Formation
10	0.5	5	No
	0.5	12.5	No
	0.5	20	Yes; beaded
	1.2	5	No
	1.2	12.5	No
	1.2	20	Yes; beaded
	2.0	5	No
	2.0	12.5	No
	2.0	20	Yes; beaded
20	0.1	22	Yes
	0.2	22	Yes; beaded
	0.3	22	Yes; beaded

## Data Availability

The original contributions presented in this study are included in the article/Supplementary Materials. Further inquiries can be directed to the corresponding author.

## Supplementary Materials

The following supporting information can be downloaded at: https://www.mdpi.com/article/10.3390/polym18131679/s1, Figure S1. Representative macroscopic appearance of kefir grains before (a) and after (b) ultrasound-assisted extraction.

## Abbreviations

The following abbreviations are used in this manuscript:

HAIs	Healthcare-associated infections
EPS	Exopolysaccharide
YPD	Yeast extract, peptone and dextrose medium
MIC	Minimum inhibitory concentration
OD	Optical density
PVA	Polyvinyl alcohol
HDFn	Primary Dermal Fibroblast Normal; Human, neonatal
MRSA	Methicillin-resistant *Staphylococcus aureus*
