# Supplementary material for: Gentamicin-Loaded Electrospun PVA/Kefiran/Schizophyllan Membrane for Skin Tissue Engineering Applications
Source: Polymers (Basel). 2026 Jul 7;18(13):1679. doi: 10.3390/polym18131679 (PMC13364103; doi:10.3390/polym18131679)
Supplement: Supplementary file 1 [file polymers-18-01679-s001.zip › polymers-4323407-supplementary.pdf]

Supplementary Figures

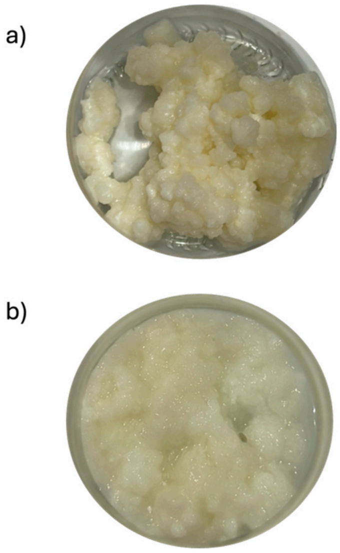

Figure S1. Representative macroscopic appearance of kefir grains before (a) and after (b) ultrasound-assisted extraction.
